# Prospective inter‐individual analysis of quality‐adjusted life years (QALYs) in brain tumor patients: A comprehensive assessment of health preferences

**DOI:** 10.1002/ijc.70171

**Published:** 2025-09-25

**Authors:** Lisa S. Hönikl, Anna Kelm, Sandro M. Krieg, Bernhard Meyer, Vicki M. Butenschoen

**Affiliations:** ^1^ Department of Neurosurgery Technical University Munich School of Medicine and Health München Germany; ^2^ Department of Neurology München Klinik Harlaching München Germany; ^3^ Department of Neurosurgery University Hospital Heidelberg Heidelberg Germany

**Keywords:** Glioblastoma Multiforme (GBM), health utilities, QALYs, quality of life

## Abstract

Eloquent intracranial tumors, whether primary or secondary, are located in brain regions critical for language, motor, or sensory function. In neuro‐oncology, evaluating treatment outcomes requires more than survival analysis alone. Quality‐adjusted life years combine survival and quality of life, based on numerical health preference values reflecting how patients perceive specific health states. However, such data are lacking for patients with eloquent tumors. In this prospective, single‐center cohort study (2016–2019), patients with eloquent brain tumors underwent standardized assessments at four time points: preoperative, postoperative, and at 3‐ and 6‐month follow‐ups. Instruments included Time Trade‐Off (TTO), Standard Gamble, EuroQol 5 Dimensions (EQ‐5D), and the Beck Depression Inventory (BDI). Patients indicated how many years from a 10‐year life span they would trade to live in perfect health, including for hypothetical deficits like hemiparesis, aphasia, or dependency. The resulting values (range 0–1) were calculated and analyzed using Mann–Whitney *U*‐tests. Preoperatively, 78 patients reported high quality of life (TTO median 0.9). EQ‐5D index declined significantly after surgery (*p* < .05), while TTO and BDI scores remained stable, reflecting a deterioration in health. At 3 months (*n* = 23), dependency was rated as less severe compared to preoperative (*p* = .055; TTO 0.2 vs. 0.5), suggesting psychological adaptation. By 6 months (*n* = 8), health perceptions improved, indicating resilience (TTO 0.7 vs. 0.6 at 3 months). This is the first study to quantify health preferences in patients with eloquent brain tumors. While postoperative health status initially declines, resilience and adaptation occur over time, emphasizing the need for tailored postoperative care.

## What's New?

Eloquent intracranial tumors present unique challenges in neurosurgical treatment due to their proximity to critical functional areas of the brain. This prospective, single‐center cohort study quantified real‐time changes in patients' subjective burden of health impairments using the Time Trade‐Off and Standard Gamble methods, providing disease‐specific data for quality‐adjusted life year calculations. The findings highlight the relevance of functional burden over tumor type and support more patient‐centered, health‐economic decision‐making in neuro‐oncology. While postoperative health status initially declined, resilience and adaptation occurred over time. These insights underscore the importance of integrating psychological adaptation and long‐term quality of life into clinical evaluations.

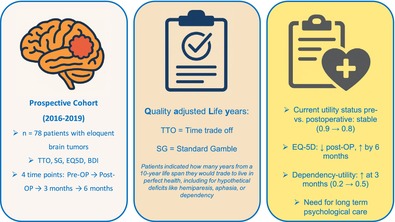

**Abbreviations**
BDI: Beck Depression Inventory
CNS: Central Nervous System
EQ‐5D: EuroQol 5 Dimensions
FUP: follow‐up
GBM: Glioblastoma Multiforme
HRQoL: health‐related quality of life
MRI: Magnetic Resonance Imaging
QALYs: quality‐adjusted life years
QoL: quality of life
SG: Standard Gamble
SIH: Spontaneous Intracranial Hemorrhage
TTO: Time Trade‐Off
VAS: Visual Analog Scale
WHO: World Health Organization

## 1 INTRODUCTION

Eloquent intracranial tumors, encompassing both primary brain tumors and metastases, present unique challenges in neurosurgical treatment due to their proximity to critical functional areas of the brain. The goal of surgery is to maximize tumor resection, which significantly improves survival^1^, ^2^ while carefully preserving neurological function, as even minor postoperative deficits can severely impair quality of life (QoL). Achieving extensive resection prolongs survival but must be balanced against the risk of neurological deterioration.^3^, ^4^ In the rapidly advancing field of neuro‐oncology, assessing treatment success based solely on survival metrics like overall survival (OS) or progression‐free survival (PFS) is increasingly seen as inadequate. Patient‐centered measures, particularly health‐related quality of life (HRQoL), are gaining prominence as critical endpoints.^5^, ^6^, ^7^, ^8^, ^9^

Especially in patients with tumors located in eloquent regions, disease experience is shaped by a complex interplay of functional impairments, emotional well‐being, social support, and individual coping strategies. Even mild deficits can drastically affect autonomy and daily living, with wide variability in how patients perceive and prioritize different health states. Personal values, resilience, and social context strongly influence these perceptions.

To meaningfully assess outcomes in this context, especially when informing treatment decisions and health policy, quality‐adjusted life years (QALYs) are often used.^10^ QALYs combine both survival duration and QoL into a single metric, enabling a more patient‐centered and nuanced evaluation of treatment benefit. Central to this approach is the concept of utility values—numerical expressions of a patient's preference for specific health states, typically ranging from 0 (the worst imaginable condition) to 1 (perfect health). In Germany, as in many other countries, utility values are rarely calculated directly for specific patient populations. This study is therefore unique in deriving these values directly from patients with eloquent brain tumors, offering a particularly patient‐centered perspective and generating insights seldom available in existing healthcare systems.

These utility values are obtained through standardized instruments such as the Time Trade‐Off (TTO) and Standard Gamble (SG) methods. Both aim to quantify the subjective burden of health impairments. Detailed descriptions of these methods and their application in this study are provided in Section 2.

Despite the potential for QALYs to inform decision‐making, there remains a significant gap in the literature regarding their application to patients with eloquent intracranial tumors.^9^, ^10^ These patients face a delicate balance between extending survival and preserving neurological function, which directly impacts their post‐treatment QoL. Furthermore, most existing studies do not address the significant inter‐individual variability in health perception and QoL outcomes that can occur depending on tumor location, histological diagnosis, neurological symptoms, and a patient's own health perception. This variability is crucial for understanding patient‐specific utility values and tailoring neurosurgical approaches to improve both survival and QoL.

This study aims to address this gap by conducting a prospective inter‐individual analysis of QoL and health preferences in patients with eloquent intracranial tumors. By comparing subgroups based on tumor type, recurrence status, and neurological deficits, the analysis highlights how different clinical characteristics influence patients' valuation of health states. Using the TTO and SG methods alongside established QoL instruments like the EuroQol 5 Dimensions (EQ‐5D) and Beck Depression Inventory (BDI), we assess how factors such as tumor type, recurrence, and neurological status impact perceived health states over time.^10^, ^11^, ^12^, ^13^ These data are essential for calculating QALYs, which serve as a key metric in assessing the cost‐effectiveness of treatments in health economics. This work seeks to enhance patient‐centered care by providing a more accurate understanding of how individuals with eloquent brain tumors perceive specific health states.

## 2 METHODS

### 2.1 Study cohort

This prospective cohort study enrolled brain tumor patients from December 2016 to December 2019 of all ages who were undergoing standard‐of‐care treatment at our institution. In these cases, standard‐of‐care treatment refers to surgical resection followed by guideline‐based adjuvant therapy depending on the underlying diagnosis, including radiotherapy and, when indicated, systemic treatment. Eligibility required patients to be willing to participate in the study and to complete a series of QoL questionnaires at four designated time points: preoperatively, postoperatively, and at a 3‐ and 6‐month follow‐up. Preoperative assessments were conducted within 7 days prior to surgery, and postoperative assessments were performed between 5 and 10 days after surgery, depending on the patient's clinical stability. Participants were recruited based on their readiness to provide informed consent and engage in the QoL assessments throughout the study period. Patients who consented to participate also had to be willing to engage with the scenario of living with conditions such as hemiparesis, dependency, or aphasia as part of the assessment process. This approach ensured a comprehensive evaluation of QoL changes associated with treatment, capturing both the immediate and longer‐term impacts on patient well‐being.

### 2.2 Study design

This study was a single‐center, prospective, non‐randomized cohort study aimed at evaluating the QoL in patients with eloquent brain tumors. Inclusion criteria for the study were as follows: adult patients (≥18 years) with a radiologically confirmed supratentorial mass lesion located in an eloquent brain region, who were scheduled to undergo neurosurgical resection and provided informed consent. Readiness to provide informed consent was assessed clinically by the treating neurosurgeon based on the patient's cognitive capacity, comprehension of the study information, and ability to communicate a voluntary decision. In cases of uncertainty regarding consent capacity, patients were not included in the study. The study design focused on capturing the real‐world impact of treatment on patients' QoL over time, without randomization, to reflect typical clinical practice settings.

To ensure the accuracy of data collection and the reliability of quality‐of‐life assessments, we excluded patients from the study if they met any of the following criteria: contraindication to Magnetic Resonance Imaging, age under 18 years, pre‐existing psychiatric disorders or dementia, or lack of informed consent to participate in the study. All patients were treated by a core team of specialized neurosurgeons within a single academic neurosurgical department, ensuring consistency in surgical approach and perioperative management. Participants were recruited consecutively based on availability and eligibility.

### 2.3 Clinical parameters

All patients underwent a physical examination at each time point (preoperatively, postoperatively, and during follow‐up). Tumor parameters, including histological type and tumor recurrence, were collected.

### 2.4 Time Trade‐Off and Standard Gamble assessment

To assess patients' health preferences and utility values, we used two established methods: TTO and SG. In the TTO assessment, patients were asked how many years out of 10 they would be willing to give up in order to live the remaining years in perfect health. For both the TTO and SG methods, we presented the patients with the following hypothetical health scenarios: hemiparesis (partial paralysis of one side of the body), aphasia (impairment in language production or comprehension), and dependency (a condition in which the patient would require assistance with daily living activities).

Both TTO and SG provide utility values ranging from 0 (equivalent to the worst imaginable health state) to 1 (perfect health) to quantify health preferences. For example, a patient facing hemiparesis might choose to live 7 years in perfect health rather than 10 years with hemiparesis, resulting in a utility score of 0.7 (calculated as (10 − 3)/10 = 0.7). This method helps quantify how patients value their QoL relative to the length of life in the presence of these conditions.

The SG method assessed patients' willingness to accept the risk of a certain condition in exchange for a chance to live in perfect health. Patients were asked to choose between living with a particular health condition (e.g., hemiparesis) or undergoing a hypothetical treatment that could result in perfect or neurological health. The utility score is derived from the probability of the patient being indifferent between the two options. For example, if a patient accepts a 40% chance of hemiparesis to be indifferent, their utility score for hemiparesis is 0.4.

### 2.5 Beck Depression Inventory and EQ‐5D assessment

The use of BDI and EQ‐5D is appropriate as they capture complementary aspects:

The BDI specifically assesses depressive symptoms, which often influence QoL and health perception, while the EQ‐5D provides a validated, generic measure of health‐related QoL, enabling standardized comparison with other populations. In addition to the utility assessments, patients completed the BDI and EQ‐5D at each evaluation point (preoperatively, postoperatively, and during follow‐up). The BDI was used to assess the severity of depressive symptoms, with scores ranging from 0 to 63, where higher scores indicate more severe depression. The EQ‐5D was employed to measure HRQoL, capturing five dimensions: mobility, self‐care, usual activities, pain/discomfort, and anxiety/depression. The EQ‐5D index score and the Visual Analog Scale (VAS) were calculated to provide a comprehensive understanding of the patient's overall health perception and QoL. These assessments, alongside the TTO and SG, offer a multidimensional view of the patients' physical and emotional well‐being throughout their treatment journey.

### 2.6 Statistics

Statistical analyses were done with R (Version 1.4.1717). Since the data did not follow a normal distribution, the Mann–Whitney *U* test was employed to assess differences between the groups. A difference with an error probability of less than .05 was considered statistically significant for all analyses. Descriptive statistics for demographic variables were generated with medians with ranges (min–max) as appropriate, along with absolute numbers and corresponding percentages.

## 3 RESULTS

### 3.1 Patient cohort

**FIGURE 1:**
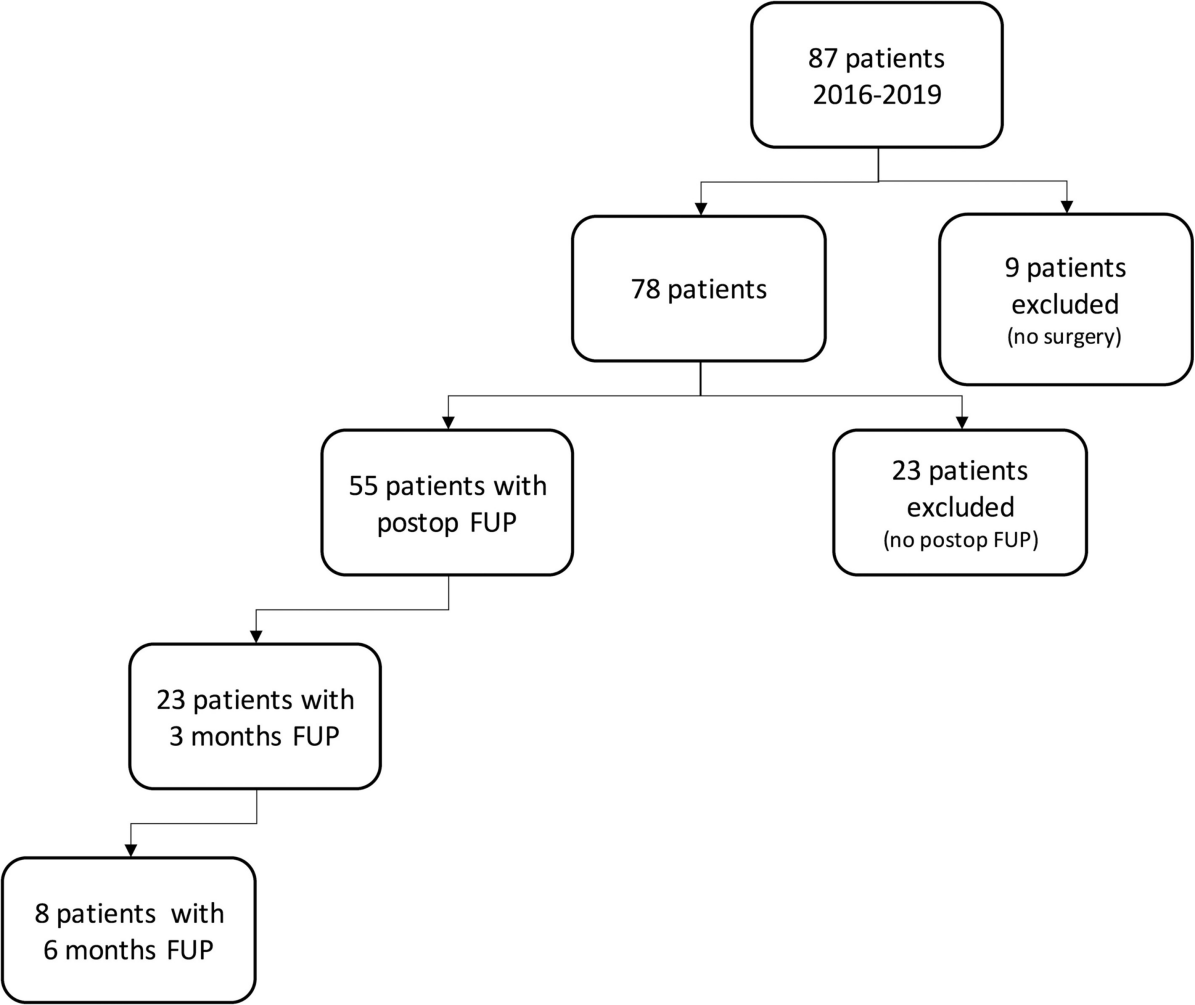
Overview of patients' cohort. FUP, follow‐up.

Between 2016 and 2019, a total of 916 patients with brain tumors, regardless of tumor type, were screened for inclusion in the study. Eighty‐seven of these 916 patients met the inclusion criteria for eloquent brain tumors and agreed to participate in the study. Of these, nine patients were excluded because they opted against undergoing surgery. Additionally, 23 patients did not participate in the postoperative follow‐up. Ultimately, 23 patients completed the 3‐month follow‐up, and eight patients participated in the 6‐month follow‐up (Figure 1).

The study included a cohort of 78 patients with cerebral lesions. The median age of the patients was 57 years, ranging from 18 to 81 years. The cohort comprised 40% females and 60% males. Among the patients, 20 (26%) presented with recurrent tumors. The histological examination of the cerebral lesions revealed a diverse range of tumor types consisting of low‐ and high‐grade gliomas (72%), particularly glioblastoma (47%), as well as metastasis (24%, Table 1). A small number of patients had meningiomas and cavernomas. One patient was initially suspected of having a tumor, but it was ultimately found to be a hemorrhage without evidence of a tumor in the histopathological examination (Table 1).

**TABLE 1:** Patient characteristics.

	Preoperative	Postoperative	FUP 3 months	FUP 6 months
Patients with cerebral lesion	78	55	23	8
Age (median [range] days)	57 (18–81)	59 (18–80)	57 (18–78)	56.5 (18–77)
Sex (female/male [%])	40/60	36/64	35/65	37.5/62.5
Recurrence (*n* [%])	20 (26)	13 (24)	8 (35)	2 (25)
Overall survival (median [range] days)	379 (6–2519)	407 (6–2519)	543 (21–2519)	437 (69–2504)
Histology				
Ganglioglioma	3	3	1	1
Pilocytic astrocytoma	1	1	1	0
Astrocytoma World Health Organization (WHO) Central Nervous System (CNS) Grade 2	5	4	4	2
Astrocytoma WHO CNS Grade 3	4	0	0	0
Oligodendroglioma WHO CNS Grade 2	3	2	1	0
Oligodendroglioma WHO CNS Grade 3	1	1	0	0
Glioblastoma WHO CNS Grade 4	37	27	10	2
Diffuse midline glioma WHO CNS Grade 4	2	0	0	0
Meningeoma WHO CNS Grade 1	1	1	0	0
Cavernoma	1	1	1	1
Prostate cancer metastasis	1	1	0	0
Adenocarcinoma metastasis	3	3	0	0
Non‐small cell lung cancer metastasis	4	2	0	0
Small cell lung cancer metastasis	1	1	1	1
Melanoma metastasis	5	5	1	0
Kidney cell metastasis	1	1	1	1
Non‐seminous germ cell tumor of the testicle metastasis	2	1	1	0
Breast cancer metastasis	2	1	1	0
Suspected tumor but bleeding caused by spontaneous intracranial hemorrhage (SIH) in the end	1	0	0	0

Abbreviation: FUP, follow‐up.

Of the 78 patients, 46% had a neurological deficit prior to surgery, including 12 (15%) with motor impairments, 12 (15%) with motor aphasia, 11 (14%) with visual field defects, and one (1%) with an oculomotor nerve palsy due to mass effect.

### 3.2 Pre‐and postoperative comparison of quality of life

**FIGURE 2:**
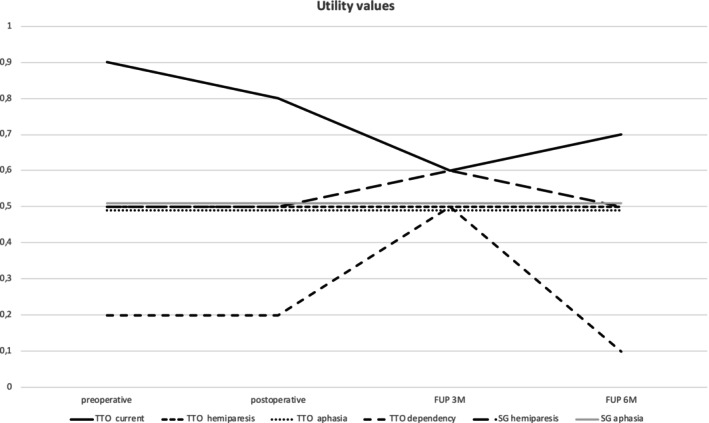
Pre‐, postoperative, follow‐up (FUP) at 3‐ and 6‐months assessment of Time Trade‐Off (TTO), Standard Gamble (SG), EQ‐5D, and Beck Depression Inventory. For TTO and SG 0 is equivalent to the worst imaginable condition and 1 represents perfect health.

Preoperative BDI scores showed a median of 7 (range 1–25), reflecting predominantly minimal to mild depressive symptoms. Postoperatively, the median BDI remained stable at 7 (range 1–18; *p* = .6895), indicating no significant change in depressive symptoms after surgery (Table 2, Figure 2).

**TABLE 2:** Pre‐ versus postoperative assessment of Time Trade‐Off (TTO), Standard Gamble (SG), EQ‐5D, and Beck Depression Inventory (BDI). For TTO and SG 0 is equivalent to the worst imaginable condition and 1 represents perfect health.

	BDI	TTO current	TTO hemiparesis	TTO aphasia	TTO dependency	SG hemiparesis	SG aphasia	EQ‐5D VAS	EQ‐5D index
Preoperative	6.5 (1–25)	0.9 (0.0–1.0)	0.5 (0.0.–1.0)	0.5 (0.0.‐1.0)	0.2 (0.0.‐1.0)	0.5 (0.0–1.0)	0.5 (0.0–1.0)	75 (0–100)	8 (5–23)
Postoperative	7 (1–18)	0.8 (0.0–1.0)	0.5 (0.0–1.0)	0.5 (0.0–1.0)	0.2 (0.0–1.0)	0.5 (0.0–1.0)	0.5 (0.0–1.0)	60 (10–90)	10 (5–29)
*p*‐Value (<.05)	.68950	.1189	.4151	.9079	.6657	.1039	.2358	.**002691**	.**03987**
FUP 3M	8 (0–21)	0.6 (0.0–1.0)	0.5 (0.0–1.0)	0.5 (0.0–1.0)	0.5 (0.0–1.0)	0.6 (0.0–1.0)	0.5 (0.0–1.0)	70 (10–100)	7 (5–15)
*p*‐Value (<.05)	.98880	.11950	.96330	.76000	.**05469**	.10790	.24890	.59350	.6242
FUP 6M	6.5 (0–21)	0.7 (0.3–1.0)	0.5 (0.2–1.0)	0.5 (0.1–1.0)	0.1 (0.0–1.0)	0.5 (0.5–1.0)	0.5 (0.2–1.0)	87.5 (50–100)	6 (5–11)
*p*‐Value (<.05)	.36850	.22580	.82630	.81160	.70620	.26710	.37470	.05165	.23190

*Note:* Bold values indicate statistically significant results (*p* < 0.05), except for *p* = 0.054 in TTO dependency, which is highlighted as it is only marginally above the threshold.

Abbreviations: FUP, follow‐up; VAS, Visual Analog Scale.

Preoperative TTO assessments revealed a high perceived QoL, with a median TTO of 0.9 (range 0.0–1.0) for the current health state. In hypothetical scenarios, median TTO values were 0.5 (range 0.0–1.0) for both hemiparesis and aphasia, and 0.2 (range 0.0–1.0) for permanent dependency, suggesting that dependency was perceived as particularly burdensome.

For the hypothetical scenarios of the occurrence of hemiparesis and aphasia, the median TTO values remained stable at 0.5 (range 0.0–1.0) postoperatively, with *p*‐values of .4151 and .9079, respectively, indicating no significant changes. The TTO value for the dependency scenario also remained stable at a median of 0.2 (range 0.0–1.0) postoperatively, with a *p* value of .6657, suggesting that patients' willingness to trade years of life to avoid dependency did not change significantly directly after surgery.

Preoperative SG values for hemiparesis and aphasia had a median of 0.5 (range 0.0–1.0), reflecting moderate risk acceptance to avoid these conditions, and remained stable after surgery (*p* = .1039 and *p* = .2358, respectively).

Regarding general health perception, the EQ‐5D VAS score was rated at a median of 75% preoperatively (range 0%–100%). Postoperatively, the median VAS significantly declined to 60% (*p* = .002691), suggesting a perceived deterioration in overall health. Similarly, the EQ‐5D index score showed a significant postoperative decrease, with a median of 10 (range 5–29) compared to the preoperative median of 8 (range 5–23; *p* = .03987).

### 3.3 Follow‐up at 3 and 6 months postoperative

At the 3‐month follow‐up (FUP 3 M), 23 patients were assessed. The median BDI score remained stable at 8 (range 0–21), with no significant change from preoperative values (*p* = .98880). The current health status assessed through TTO showed a slight, statistically non‐significant decrease to 0.6 (range 0.0–1.0, *p* = .11950). TTO values for hypothetical conditions, such as hemiparesis (0.5 [0.0–1.0], 0.96330) and aphasia (0.5 [0.0–1.0], 0.76000) remain consistent with preoperative values. The TTO value for dependency (0.5 [0.0–1.0], 0.05469) was notably higher during the 3‐month follow‐up compared to the preoperative, immediate postoperative, and 6‐month follow‐up assessments. This indicates that at the 3‐month follow‐up, patients perceived the state of dependency as less burdensome, as reflected by a higher TTO value, meaning they were less inclined to trade life years to achieve perfect health compared to the preoperative, immediate postoperative, and 6‐month assessments. Despite the small sample size, a clear trend emerged, suggesting a shift in patient perception, although this difference narrowly missed statistical significance. The SG values also showed no significant changes for hemiparesis (0.6 [0.0–1.0], 0.10790) and aphasia (0.5 [0.0–1.0], 0.24890). The EQ‐5D VAS score decreased to a median of 70 (range 10–100), though this change was not statistically significant (*p* = .59350), and the EQ‐5D index score remained stable (Table 2).

At the 6‐month follow‐up (FUP 6 M), data from eight patients were analyzed. The median BDI score slightly decreased to 6.5 (range 0–21), equal to the preoperative value. The current health state assessed through TTO showed a median of 0.7 (range 0.3–1.0), but this change was not statistically significant (*p* = .26210). TTO values for hypothetical conditions, such as hemiparesis (0.5 [0.2–1.0], 0.82630), aphasia (0.5 [0.1–1.0], 0.81160), and dependency (0.1 [0.0–1.0], 0.70620), remained consistent with preoperative values. The SG values also showed no significant changes for hemiparesis (0.5 [0.5–1.0], 0.26710) and aphasia (0.5 [0.2–1.0], 0.37470). The EQ‐5D VAS score slightly increased to a median of 87.5 (range 50–100), approaching significance (*p* = .05165), suggesting a potential improvement in perceived health status at this later stage. Overall, the follow‐up data indicate relative stability in most QoL measures, with some variations observed in specific domains over time (Table 2). A notable finding is that both the 3‐ and 6‐month follow‐up cohorts mainly included patients with lower EQ‐5D scores. At 3 months, patients who remained in follow‐up had a median EQ‐5D score of 10, compared to 14 in those lost to follow‐up. This suggests that patients with greater perceived health impairments were more likely to continue participating, potentially introducing a bias toward worse reported outcomes during follow‐up.

### 3.4 Differences in assessment between patients with initial diagnosis and recurrence

In our cohort, 26% of patients presented with tumor recurrence at enrollment. Patients with newly diagnosed tumors had a median preoperative BDI score of 8 (range 1–25), compared to 5 (range 2–19) in recurrent cases. This difference was not statistically significant (*p* = .2927), indicating similar levels of depressive symptoms between groups (Table 3).

**TABLE 3:** Comparison between newly diagnosed and recurrence tumors of Time Trade‐Off (TTO), Standard Gamble (SG), EQ‐5D, and Beck Depression Inventory (BDI). For TTO and SG 0 is equivalent to worst imaginable condition and 1 represents perfect health.

	BDI	TTO current	TTO hemiparesis	TTO aphasia	TTO dependency	SG hemiparesis	SG aphasia	EQ‐5D VAS	EQ‐5D index
Newly diagnosed	8 (1–25)	0.83 (0.0–1.0)	0.5 (0.0–1.0)	0.5 (0.0–1.0)	0.15 (0.0–1.0)	0.5 (0.0–1.0)	0.5 (0.0–1.0)	77.5 (0–100)	8 (5–23)
Recurrence	5 (2–19)	1.0 (0.3–1.0)	0.5 (0.0–1.0)	0.5 (0.0–1.0)	0.2 (0.0–1.0)	0.5 (0.5–1.0)	0.45 (0.0–1.0)	75 (20–100)	6.5 (5–20)
*p*‐Value (<.05)	.2927	.3279	.6338	.1547	.6918	.5069	.5612	.8991	.2073

Abbreviation: VAS, Visual Analog Scale.

The preoperative TTO score for current health was slightly lower in newly diagnosed patients (median 0.83, range 0.0–1.0) compared to recurrent cases (median 1.0, range 0.3–1.0), though not statistically significant (*p* = .3279). This may suggest greater resilience in patients living longer with the disease. For hypothetical scenarios, TTO values for hemiparesis and aphasia were identical between groups (median 0.5). TTO scores for dependency were similar as well (0.15 vs. 0.2; *p* = .6918), indicating no significant difference (Table 3).

The SG values for the scenario of hemiparesis were consistent in both groups, with a median of 0.5 (range 0.0–1.0) for newly diagnosed patients (*n* = 46) and a similar median of 0.5 (range 0.5–1.0, *n* = 15) for those with recurrence. The *p*‐value of .5069 indicates no significant difference. For aphasia, the SG median was slightly lower in patients with recurrent tumors at 0.45 (range 0.0–1.0, *n* = 14) compared to 0.5 (range 0.0–1.0, *n* = 46) in newly diagnosed patients, but again, this difference did not reach statistical significance (*p* = .5612, Table 3).

The EQ‐5D VAS showed a median of 77.5 (range 0–100, *n* = 52) for newly diagnosed patients, compared to a median of 75 (range 20–100, *n* = 17) for those with recurrence, with no significant difference between the groups (*p* = .8991). Similarly, the EQ‐5D index score was 8 (range 5–23, *n* = 51) for newly diagnosed patients and 6.5 (range 5–20, *n* = 16) for patients with recurrence, with a *p*‐value of .2073, indicating no significant difference in the overall health‐related QoL between the two groups (Table 3).

### 3.5 Comparison between patients with and without preoperative neurological deficits

Among the 78 patients, 46% presented with a preoperative neurological deficit, including 15% with hemiparesis and 15% with motor aphasia. The remaining 16% of patients presented with other neurological deficits, including visual field impairments, cranial nerve dysfunctions, or altered levels of consciousness. Patients without deficits had a slightly higher median BDI score (7, range 1–25) compared to those with deficits (6, range 1–19), but the difference was not statistically significant (*p* = .8167).

For the current health state, patients without neurological deficits reported a higher median TTO value of 1.0 (range 0.1–1.0, *n* = 37) compared to 0.83 (range 0.0–1.0, *n* = 26) in those with deficits, although this difference did not reach statistical significance (*p* = .12360). The TTO values for hypothetical conditions such as hemiparesis, aphasia, and dependency showed only minor differences between the groups, with no significant disparities. Patients with preoperative hemiparesis had a median TTO value of 0.5 for the hemiparesis scenario, compared to 0.5 for patients without hemiparesis (*p* = .8751). Patients with preoperative aphasia had a median TTO value of 0.2 (range 0.0–1.0) for the aphasia scenario, compared to 0.5 (range 0.0–1.0) for those without aphasia (*p* = .4636). Patients with preoperative aphasia perceived the condition of aphasia as more severe compared to patients without aphasia, but again, without reaching statistical significance.

In terms of risk perception, SG values for hemiparesis and aphasia were slightly higher in patients without deficits, with a median of 0.6 (range 0.0–1.0, *n* = 35) and 0.55 (range 0.0–1.0, *n* = 34), respectively, compared to 0.5 (range 0.0–1.0) for both scenarios in patients with neurological deficits. However, these differences were not statistically significant. Patients with preoperative hemiparesis had a median SG value of 0.4 for the hemiparesis scenario, compared to 0.5 for patients without hemiparesis (*p* = .125). Similarly, patients with preoperative aphasia had a median SG value of 0.4 (range 0.0–1.0) for the aphasia scenario, compared to 0.5 (range 0.0–1.0) for those without aphasia (*p* = .3907).

The EQ‐5D VAS score was higher in patients without neurological deficits, with a median of 80 (range 30–100, *n* = 41), compared to 70 (range 0–95, *n* = 28) in those with deficits, though the *p*‐value of .19850 indicates this difference was not statistically significant. The EQ‐5D index score showed a trend toward significance (*p* = .05152), with a higher median score in patients without deficits (7 vs. 8), suggesting that those without preoperative deficits perceived their health‐related QoL more favorably.

Overall, while patients with and without preoperative neurological deficits reported similar scores across most measures, those without deficits understandably rated their health more positively—particularly regarding their current health state and overall QoL—reflecting the expected impact of preserved neurological function (Table 4).

**TABLE 4:** Comparison between patients with and without neurological deficits of Time Trade‐Off (TTO), Standard Gamble (SG), EQ‐5D, and Beck Depression Inventory (BDI). For TTO and SG 0 is equivalent to worst imaginable condition and 1 represents perfect health.

	BDI	TTO current	TTO hemiparesis	TTO aphasia	TTO dependency	SG hemiparesis	SG aphasia	EQ‐5D VAS	EQ‐5D index
No neurological deficit	7 (1–25)	1.0 (0.1–1.0)	0.5 (0.0–1.0)	0.5 (0.0–1.0)	0.1 (0.0–1.0)	0.6 (0.0–1.0)	0.55 (0.0–1.0)	80 (30–100)	7 (5–18)
Neurological deficit	6 (1–19)	0.83 (0.0–1.0)	0.6 (0.0–1.0)	0.5 (0.0–1.0)	0.28 (0.0–1.0)	0.5 (0.0–1.0)	0.5 (0.0–1.0)	70 (0–95)	8 (5–23)
*p*‐Value (<.05)	.81670	.12360	.81670	.81670	.58930	.08902	.48000	.19850	.**05152**

*Note:* EQ‐5D index was highlighted in bold because the value of *p* = 0.051 is only slightly above the 0.05 threshold and was therefore emphasized.

Abbreviation: VAS, Visual Analog Scale.

### 3.6 Differences between gliomas and metastases

Patients with gliomas and those with metastases exhibited comparable outcomes across multiple measures (Table 5). The median BDI score for glioma patients was 6 (range 1–25), while for patients with metastases it was 8 (range 1–18; *p* = .9621). For the TTO assessments, the median value for the current health state was 0.9 in both groups (gliomas: range 0.1–1.0; metastases: range 0.0–1.0; *p* = .9440), and the median TTO values for hemiparesis were identical at 0.5 (range 0.0–1.0; *p* = .9666). The median TTO for aphasia was 0.5 for gliomas compared to 0.6 for metastases (*p* = .8222), while for dependency the values were 0.1 versus 0.2, respectively (*p* = .7699). Similarly, SG values for hemiparesis were 0.6 in gliomas and 0.5 in metastases (*p* = .2526), and for aphasia 0.6 versus 0.4 (*p* = .1945). Although the EQ‐5D VAS was higher in glioma patients (median 80 vs. 65; *p* = .1549), and the EQ‐5D index was identical (median 11; *p* = .8531), none of these differences reached statistical significance, suggesting that the impact on QoL is similar regardless of tumor type.

**TABLE 5:** Ccomparison between gliomas and metastases of preoperative Time Trade‐Off (TTO), Standard Gamble (SG), EQ‐5D, and Beck Depression Inventory (BDI). For TTO and SG 0 is equivalent to worst imaginable condition and 1 represents perfect health.

	BDI	TTO current	TTO hemiparesis	TTO aphasia	TTO dependency	SG hemiparesis	SG aphasia	EQ‐5D VAS	EQ‐5D index
Gliomas	6 (1–25)	0.9 (0.1–1.0)	0.5 (0.0–1.0)	0.5 (0.0–1.0)	0.1 (0.0–1.0)	0.6 (0.0–1.0)	0.6 (0.0–1.0)	80 (0–100)	11 (5–25)
Metastasis	8 (1–18)	0.9 (0.0–1.0)	0.5 (0.0–1.0)	0.6 (0.0–1.0)	0.2 (0.0–1.0)	0.5 (0.0–1.0)	0.4 (0.0–1.0)	65 (30–95)	11 (5–28)
*p*‐Value (<.05)	.9621	.9440	.9666	.8222	.7699	.2526	.1945	.1549	.8531

Abbreviation: VAS, Visual Analog Scale.

## 4 DISCUSSION

The findings of this study provide valuable insights into the QoL and health preferences of patients with eloquent intracranial tumors, both preoperatively and postoperatively, as well as between newly diagnosed cases and those with tumor recurrence and patients with and without preoperative neurological deficits. By evaluating QoL through various measures, including the BDI, TTO, SG, and EQ‐5D, we were able to capture a comprehensive picture of how these patients perceive their individual health and make trade‐offs between quality and quantity of life. An important consideration when interpreting our findings is the substantial loss of participants during follow‐up, which may have introduced bias, as patients lost to follow‐up are likely not missing at random; this limitation is addressed in more detail below.

### 4.1 Pre‐ and postoperative outcomes

The stability of BDI scores from the preoperative to the postoperative period suggests that depressive symptoms remained relatively unchanged directly following surgery. This may indicate that the surgical intervention, while crucial for disease management, did not exacerbate depressive symptoms within the first days after surgery.^14^ Depression is known to occur in approximately 17% of patients within a year following surgery,^15^, ^16^ a trend that is similarly observed in our study. Initially, depression scores did not significantly increase postoperatively; however, a noticeable rise was seen at the 3‐month follow‐up, followed by an improvement at 6 months. This pattern suggests an initial psychological impact post‐surgery, with potential for recovery and adaptation over time.

The TTO values indicated that patients generally maintained a high valuation of their life postoperatively, particularly concerning their general health and current health state. This consistency in TTO scores suggests that the patients' perception of their health quality did not drastically decline following surgery, which is encouraging for the overall effectiveness of the intervention. However, the slight decrease in the TTO score for the current health state postoperatively, though not statistically significant, may reflect the physical and emotional recovery challenges patients face after surgery.

SG values, used to assess patients' willingness to accept risk to avoid conditions like hemiparesis and aphasia, remained stable postoperatively. This stability might indicate that patients' risk tolerance concerning potential neurological deficits remains consistent before and after surgery, highlighting the importance of preoperative counseling and setting realistic expectations about surgical outcomes.

The EQ‐5D VAS showed a significant decrease postoperatively, suggesting that patients perceived a decline in their overall health status after surgery. This finding is critical, as it points to the need for enhanced postoperative care and support to address patients' perceptions of their health and possibly improve their long‐term outcomes. Furthermore, this pattern aligns with what is observed in the literature from other types of surgical procedures, where an initial postoperative decline in EQ‐5D scores is common, followed by gradual improvements over time as patients recover.^11^, ^17^, ^18^, ^19^ The significant drop in the EQ‐5D index score further supports this, indicating that patients experienced a tangible reduction in their health‐related QoL following surgery.

### 4.2 Follow‐up outcomes

The follow‐up results at 3 and 6 months postoperatively reveal important insights into the trajectory of patients' QoL and health perceptions after surgery for eloquent intracranial tumors. The stability of BDI scores and the consistent TTO and SG values across the follow‐up periods suggest that patients' depressive symptoms and their willingness to trade years of life for health remained largely unchanged. However, the significant decrease in the EQ‐5D VAS score at 3 months, followed by a noticeable, though not statistically significant, improvement at 6 months, may indicate a period of adjustment and adaptation following surgery. This pattern is suggestive of posttraumatic growth—a psychological phenomenon where individuals, after experiencing trauma or significant stress, report positive psychological change.^20^, ^21^, ^22^, ^23^ The initial decline in perceived health status at 3 months could reflect the immediate physical and emotional challenges post‐surgery, while the subsequent improvement at 6 months could indicate a phase of recovery where patients begin to reframe their experience, find new coping mechanisms, and potentially experience growth. This trend is further supported by the TTO value for dependency, which was notably higher at 0.5 during the 3‐month follow‐up compared to preoperative and immediate postoperative assessments, suggesting a shift in how patients perceive dependency over time as they adapt to their health condition, even though this difference narrowly missed statistical significance. Interestingly, the slight decline in this value at 6 months may reflect a selection effect: patients who remained in follow‐up at this later stage may have been in better overall health and thus perceived the prospect of dependency more critically or with greater concern, given their regained sense of independence.

### 4.3 Preoperative neurological deficits

In contrast, the comparison between patients with and without preoperative neurological deficits highlights how the presence of pre‐existing deficits can shape perceptions of health and QoL. Patients without neurological deficits generally reported higher EQ‐5D VAS and index scores, suggesting a more favorable view of their health preoperatively. Although these differences were not statistically significant, the trend suggests that patients who enter surgery with intact neurological function may feel more resilient or optimistic about their health outcomes. On the other hand, patients with preoperative deficits might already perceive their health as compromised, which could influence their overall QoL assessments and make them less likely to report significant improvements postoperatively.

### 4.4 Newly diagnosed versus recurrence

When comparing patients with newly diagnosed tumors to those with recurrent tumors, the results revealed no significant differences in BDI scores, TTO, SG values, or EQ‐5D scores. This finding is somewhat surprising, as one might expect that patients with recurrent tumors, who are likely more aware of the challenges posed by the disease, might report different QoL outcomes. The lack of significant differences may suggest that the psychological and health‐related QoL impacts of a first diagnosis are as profound as those experienced by patients facing a recurrence. This highlights the importance of robust support systems, resilience of the patient, and comprehensive care plans for all patients, regardless of whether they are newly diagnosed or experiencing a recurrence.^24^, ^25^, ^26^

The TTO scores were slightly higher for patients with recurrent tumors when assessing their current health state, although this was not statistically significant. This may indicate a subtle difference in how these patients value their remaining time, potentially due to a more acute awareness of their mortality.^20^, ^27^, ^28^ The SG values for hemiparesis and aphasia were similar across both groups, reinforcing the idea that the perceived severity of these potential outcomes remains consistent, regardless of whether the patient is facing a first diagnosis or a recurrence.

The EQ‐5D VAS and index scores did not differ significantly between the two groups, suggesting that both newly diagnosed and recurrent patients perceive their health status similarly. This finding is important for clinical practice, as it indicates that interventions aimed at improving QoL should be applied consistently across both groups.

### 4.5 Differences between gliomas and metastases

To increase the study's sample size and statistical power, both gliomas and metastases were included, as our primary focus was on tumor localization within eloquent brain areas rather than the specific histological diagnosis. One might expect that patients with metastases, having potentially experienced their disease for a longer period, would be more accustomed to their condition and thus exhibit different utility scores compared to glioma patients. However, our analysis revealed no significant differences in QoL outcomes or utility measures between these two groups. This finding may indicate that the challenges in preserving neurological function—and consequently, patients' health perceptions—are more closely linked to the tumor's location than its diagnosis. Alternatively, the absence of differences could be attributed to the small number of metastasis cases and potential selection bias, as only those with particularly prominent brain lesions were included. Further research with larger, more homogeneous cohorts is warranted to explore any subtle differences that may exist between these patient groups.

### 4.6 Differences between brain tumors and other diseases

Studies using TTO and SG in stroke populations have found that major neurological deficits yield very low utility values—on par with those seen in eloquent brain tumor patients. For example, a post‐stroke hemiparesis (one‐sided paralysis) is associated with a mean utility on the order of 0.2–0.3, which plummets toward ~0.1 when accompanied by severe aphasia.^29^ Such values are comparable to those reported in patients with eloquent brain tumors experiencing similar motor or language deficits. In both cases, these utility scores indicate a drastic reduction in QoL. Furthermore, being fully dependent in daily activities—often the outcome of a severe stroke—is rated at or below zero (i.e., “worse than death”) on utility scales.^30^ Eloquent glioma patients with profound disabilities likewise report extremely low utilities, underscoring that hemiparesis, aphasia, and dependency have similarly devastating impacts on life quality across stroke^29^, ^30^ and brain tumor conditions.

## 5 LIMITATIONS

This study has several limitations. Firstly, the relatively small sample size, particularly within subgroups such as recurrent tumors, may limit the statistical power to detect subtle differences. Nevertheless, it remains the only series to date that prospectively quantified health preferences using both TTO and SG methods in neurosurgical patients, offering critical insights for QALY‐based evaluations. A significant number of patients were excluded due to tumor location, while others declined participation due to the demanding study protocol or the emotional burden of confronting hypothetical scenarios. Secondly, as a single‐center study, the generalizability of the findings is limited. Thirdly, the short follow‐up period restricts insights into long‐term outcomes, and the reliance on self‐reported measures like TTO, SG, EQ‐5D, and BDI introduces potential subjective biases. Variability in tumor types and neurological status further complicates specific interpretations. A notable limitation is the considerable loss of participants during follow‐up. Given the clinical vulnerability of this patient population, attrition is expected; however, it is unlikely to be random. Patients with poorer perceived health may have been less likely to continue, potentially biasing the results toward more favorable outcomes. Conversely, it is also possible that those with greater health impairments were more motivated to attend follow‐up visits, introducing bias also in the opposite direction.

Postoperative anesthesia effects and patients' memory of previous assessments could have influenced responses. Furthermore, hypothetical constructs like future health states (e.g., dependency or aphasia) are inherently difficult to assess accurately, though such approaches are consistent with prior studies.^31^

Finally, differences between primary and secondary tumors and the potential influence of adjuvant treatments as well as the social environment were not specifically addressed, representing further areas for future research.

## 6 CONCLUSION

This study uniquely quantifies health preferences in patients with eloquent intracranial tumors using TTO and SG methods, providing essential data for QALY calculations in neuro‐oncology. Although depressive symptoms and overall life valuation remained stable after surgery, patients reported a significant deterioration in health status, reflected by decreased EQ‐5D scores. Interestingly, dependency was perceived as less severe 3 months postoperatively, suggesting temporary psychological adaptation. QoL outcomes were similar regardless of tumor type or recurrence, indicating that neurological impact is primarily driven by functional status rather than histology. These findings highlight the need for comprehensive postoperative care focusing on physical rehabilitation and psychological support to foster resilience. Future strategies should aim to mitigate early declines in perceived health and improve long‐term outcomes.

## AUTHOR CONTRIBUTIONS

**Lisa S. Hönikl:** Writing – original draft; formal analysis; visualization; writing – review and editing. **Anna Kelm:** Writing – review and editing; formal analysis; data curation. **Sandro M. Krieg:** Conceptualization; writing – review and editing; supervision. **Bernhard Meyer:** Supervision; writing – review and editing. **Vicki M. Butenschoen:** Conceptualization; data curation; formal analysis; writing – review and editing; supervision; project administration. Critical revision for important intellectual content: all authors. Final approval: all authors. **Vicki M. Butenschoen** and **Sandro M. Krieg** agreed to be accountable for all aspects of the work to ensure that questions related to the accuracy or integrity of any part of the work are appropriately investigated and resolved.

## CONFLICT OF INTEREST STATEMENT

All authors report no conflict of interest concerning the materials or methods used in this study or the findings specified in this publication.

## ETHICS STATEMENT

The presented study meets the ethical standards outlined in the Declaration of Helsinki, ethics approval was obtained, and the favorable vote was registered under the number 306/16S. In this prospective study, informed consent for study participation was signed by each patient.

## Data Availability

The datasets used and/or analyzed during the current study are available from the corresponding author on reasonable request. R code is publicly available on GitHub (https://github.com/lhoenikl/QALY-analysis.git).
